# Impact of provider-selected indication requirement on urine test utilization and positivity

**DOI:** 10.1017/ash.2022.243

**Published:** 2022-06-23

**Authors:** Jessica A. Penney, Angie Mae Rodday, Paola Sebastiani, David R. Snydman, Shira I. Doron

**Affiliations:** 1 Division of Geographic Medicine and Infectious Disease, Tufts Medical Center, Boston, Massachusetts; 2 Institute for Clinical Research and Health Policy Studies, Tufts Medical Center, Boston, Massachusetts

## Abstract

**Objective:**

To evaluate the impact of the addition of an indication specification requirement to isolated urine-culture ordering on testing utilization.

**Design:**

Retrospective study utilizing interrupted time series analysis with negative binomial regression. The preintervention period was October 1, 2018–November 11, 2019, and the postintervention period was November 12, 2019–October 31, 2020. The primary outcome was isolated culture rate per 1,000 patient days. Secondary outcomes were the proportion of all urine tests ordered as isolated urine culture and culture positivity. An exploratory analysis assessed the appropriateness of selected testing indications.

**Setting:**

A 415-bed, urban, academic medical center.

**Patients:**

Adult patients with urine testing performed during hospital admission. In total, 1,494 unique isolated urine-culture orders were included in the analysis.

**Interventions:**

On November 12, 2019, the laboratory order interface was changed to require the ordering provider to select an indication for isolated urine culture.

**Results:**

Isolated urine-culture rates did not significantly change after the intervention (11.2–7.8 cultures per 1,000 patient days; *P* = .17) nor did culture positivity (26.9% vs 26.8%). Most ordering providers left the indication for testing blank, and of those charts reviewed, 67% did not have a documented condition for which isolated urine culture was the most appropriate initial test.

**Conclusions:**

The addition of an order-specification requirement for isolated urine-culture testing did not significantly affect ordering practices. The test remains overused as the initial diagnostic evaluation for a suspected urinary tract infection. Further provider education and continued changes in provider workflow are needed to achieve lasting change in practice.

Urinary tract infections (UTIs) are a common reason for healthcare visits, with >100,000 hospitalizations annually.^1^ Accurately diagnosing an infection is critical to appropriately managing patients. Urine testing should be ordered in response to symptoms of a UTI but is instead often ordered for nonspecific indications, such as fever, leukocytosis, or altered mental status, resulting in substantial use of laboratory testing materials and hospital costs.^2^ When cultures are positive, even if the patient lacks symptoms of a UTI, antibiotics are frequently prescribed. This situation contributes to overuse of antibiotics^3,4^ and growing antimicrobial resistance.^5,6^

Antibiotic prescription for asymptomatic bacteriuria, as defined by the presence of significant bacterial colony count (>10^5^ colony-forming units (CFU)/mL of 1 bacterial species) on urine culture without symptoms of UTI,^7^ is not recommended for most patients.^8^ Current guidelines^8,9^ suggest that asymptomatic bacteriuria should not be screened for or treated except for particular patient groups such as those who are pregnant, those undergoing urologic procedures that damage the mucosa, and those with profound neutropenia.

Diagnostic testing stewardship, which is the optimization of the process of ordering, performing, and reporting diagnostic tests, has been used in many institutions to address the issues of inappropriate testing and the resulting antibiotic overprescribing.^10^ Stewardship has been shown to decrease false-positive results, resulting in fewer iatrogenic adverse events and shorter hospital stays.^10^ An example of diagnostic testing stewardship in urine testing is the use of algorithms including the “urinalysis with reflex to culture” (UARC).^11–16^ When a urine specimen is ordered as UARC, a urinalysis is performed and the specimen is only processed for culture if certain criteria are met that suggest the possibility of infection. The UARC is not the most appropriate test for all patients. For some patient cultures without preceding urinalysis, isolated urine-culture testing is indicated, including those special patient groups in whom screening and treatment of asymptomatic bacteriuria is recommended. However, isolated urine-culture testing is often overused.^17^ It is frequently ordered for patients in whom UARC is the most appropriate initial diagnostic test, contributing to overtreatment of patients with asymptomatic bacteriuria.

We first implemented UARC at our academic medical center in 2014, leaving 3 options for urine testing: urinalysis with reflex to culture, urinalysis alone, or isolated urine culture. The preferred test is UARC for most patients. In this study, we evaluated the impact of the addition of an indication selection requirement to the laboratory order interface for isolated urine-culture testing on culture rates and subsequent culture positivity as a diagnostic stewardship intervention to guide the appropriate use of this test. We found that urine culture was overutilized in hospitalized patients. We also evaluated the appropriateness of selected indications for testing after the intervention was implemented. The findings of this study can potentially inform diagnostic practices for urine testing at our healthcare institution and elsewhere.

## Methods

### Study setting

This retrospective study included patients admitted to Tufts Medical Center (TMC) between December 1, 2018, and October 31, 2020, who had urine testing ordered during their admission. Tufts Medical Center is a 415-bed, academic medical center in Boston, Massachusetts. For patients with multiple tests ordered during their admission, only the initial test was included in the study. We excluded patients aged <18 years and patients who had testing performed in an outpatient setting. The selection process for study inclusion is described in the Supplementary Figure 1). This study was approved by Tufts Health Sciences Institutional Review Board and was granted exempt status.

### Data collection

Patient and laboratory data were abstracted from the hospital laboratory ordering database. Data included patient demographic characteristics (age and sex), microbiology results, and selected indication for isolate urine-culture testing (only available on postintervention samples). Information regarding patient days was abstracted from data collected by the TMC infection prevention department. Appropriateness of testing was assessed by independent chart review (performed by J.P.).

### Definitions

Urine cultures with growth of an organism >100,000 colony-forming units per milliliter (CFU/mL) were considered positive. Cultures that were negative for significant growth (<100,000 CFU/mL), grew mixed flora, or were contaminated were considered negative. Appropriateness of testing on chart review was defined as meeting clinical criteria for use of the isolated urine culture order (listed below) as the initial test for infection.^8,9^

### Intervention

The addition of an indication requirement with the isolated urine culture order in the electronic medical record (EMR) was implemented on November 12, 2019. Indication options for providers consisted of the clinically appropriate indications for isolated urine culture: neutropenia, pregnancy, before urologic surgery, sample volume inadequate to perform urinalysis (<1 mL) in preterm neonates only, nephrologist has already performed point-of-care urinalysis, “none of the above” (meaning the lack of a clinical indication for isolated urine culture), and “other” (meaning another perceived indication for testing). The order interface also allowed providers to leave the selected indication blank and continue with order placement. Throughout both the pre- and postintervention periods, the infection prevention and antimicrobial stewardship teams conducted educational interventions focused on appropriate diagnostic testing, although capacity was diminished for educational initiatives during the coronavirus disease 2019 (COVID-19) preparedness and initial pandemic periods (January 1, 2020–October 31, 2020).

### Outcomes

The primary outcome was the change in isolated urine-culture rates per 1,000 patient days between the preintervention period (December 1, 2018–October 31, 2019) and the postintervention period (November 1, 2019–October 31, 2020). Additional outcomes assessed were measures of testing utilization including the change in culture positivity (proportion of isolated urine cultures with bacterial growth) and isolated urine-culture orders as a proportion of all urine testing (which includes both UARC and isolated urine culture). In an exploratory analysis, selected provider indications for isolated culture testing after the intervention were collected and analyzed. A random subset of patients was selected for chart review to evaluate for appropriateness of isolated culture testing and agreement between selected indication and indication on chart review.

### Statistical analysis

Sample size was calculated for the primary outcome: the urine-culture rate. As an estimate for the interrupted time series analysis, sample size and power were calculated for an analysis of covariance (ANCOVA). A difference of 2.5 cultures per 1,000 patients between study periods was utilized for the estimate, corresponding to an effect size of 0.1 (SD, 25). Assuming a 2-sided α of .05 and power of 80%, the required sample size was 787 cultures overall.

Patient characteristics in the overall study period were reported, and comparisons of the intervention periods were made using the Student *t* test and the χ^2^ test. Primary and secondary outcomes by month were summarized using median with interquartile ranges (IQRs), and comparisons of the intervention periods were made using Wilcoxon rank-sum testing. For the primary analysis, an interrupted time series analysis^18^ with negative binomial regression was performed to estimate the change in outcomes, as well as to assess for the pre- and postintervention trends. Due to overdispersion, negative binomial regression was used rather than Poisson regression. Graphical representation of the interrupted time series analysis utilized regression lines with Newey-West standard errors with lag(0). A *P* value of <.05 was considered statistically significant. Additional exploratory analysis was performed on postintervention test data to determine frequency of selected indications. A subset 100 patients was selected for chart review to assess agreement between selected indication for testing and indication on chart review. The study period included the onset of the COVID-19 pandemic, so we used a sensitivity analysis excluding March–May 2020 because this period had the greatest volume of COVID-19 patients admitted to the institution and elective procedures were suspended during this time. All statistical analyses were performed using R studio version 4.1.1717 software (R Core Team, Vienna, Austria). Plots of the interrupted time series analysis were created utilizing Stata version 16.1 software (StataCorp, College Station, TX).

## Results

### Patient characteristics

During the study period, 1,494 isolated urine cultures were ordered: 891 cultures were ordered before the intervention and 603 cultures were ordered after the intervention. We detected no significant differences in age of patients for whom testing was performed. However, we detected a significant difference in patient sex between the intervention periods with fewer males having isolated urine culture performed in the postintervention period (Table 1).

**Table 1.** Patient Characteristics during the Pre- and Postintervention Periods

Characteristic	Study Cohort (N=1,494)	Preintervention Period (N=891)	Postintervention Period (N=603)	*P* Value^a^
Age, median y (IQR)	62.9 (49.0–74.8)	63.3 (50.5–75.7)	61.7 (45.4–73.9)	.18
**Sex, No. (%)**				<.001
Female	735 (49.2)	396 (44.4)	339 (56.2)	
Male	759 (50.8)	495 (55.6)	264 (43.8)	

Note. IQR, interquartile range.

a Compared via Student *t* test or χ^2^ test where appropriate.

### Isolated culture characteristics

Our initial analysis included comparison of medians for the primary outcomes summarized at the monthly level (Table 2). We detected a significant decrease in rates of isolated urine culture testing, as well as in the proportion that isolated urine cultures represented among all tests performed. There was no change in isolated urine culture positivity (25.6% preintervention vs 25.2% postintervention).

**Table 2.** Isolated Urine Culture Characteristics during the Pre- and Postintervention Periods

Variable	Study Cohort, (N=1,494)	Before the Intervention, (N=891)	After the Intervention, (N=603)	*P* Value^a^
Isolated culture rate, median % (IQR)^b^	8.9 (6.9–9.6)	9.5 (8.9–10.7)	6.9 (6.2–7.9)	<.001
Proportion of all urine testing, median % (IQR)^c^	11.6 (8.9–11.5)	13.4 (12.1–15.2)	8.9 (8.1–10.5)	<.001
Culture positivity, median % (IQR)^d^	25.6 (20.6–27.3)	25.6 (20.9–26.7)	25.2 (20.5–29.2)	.69

Note. Data analyzed on month level. IQR, interquartile range; UARC, urinalysis with reflex to culture.

a Compared via Wilcoxon rank-sum testing.

b Isolated culture rate per 1,000 patient days.

c Proportion of all urine testing measured as isolated urine culture/all urine testing (isolated urine culture + UARC).

d Culture positivity measured as culture positive for organisms/isolated culture performed.

**Fig. 1.**
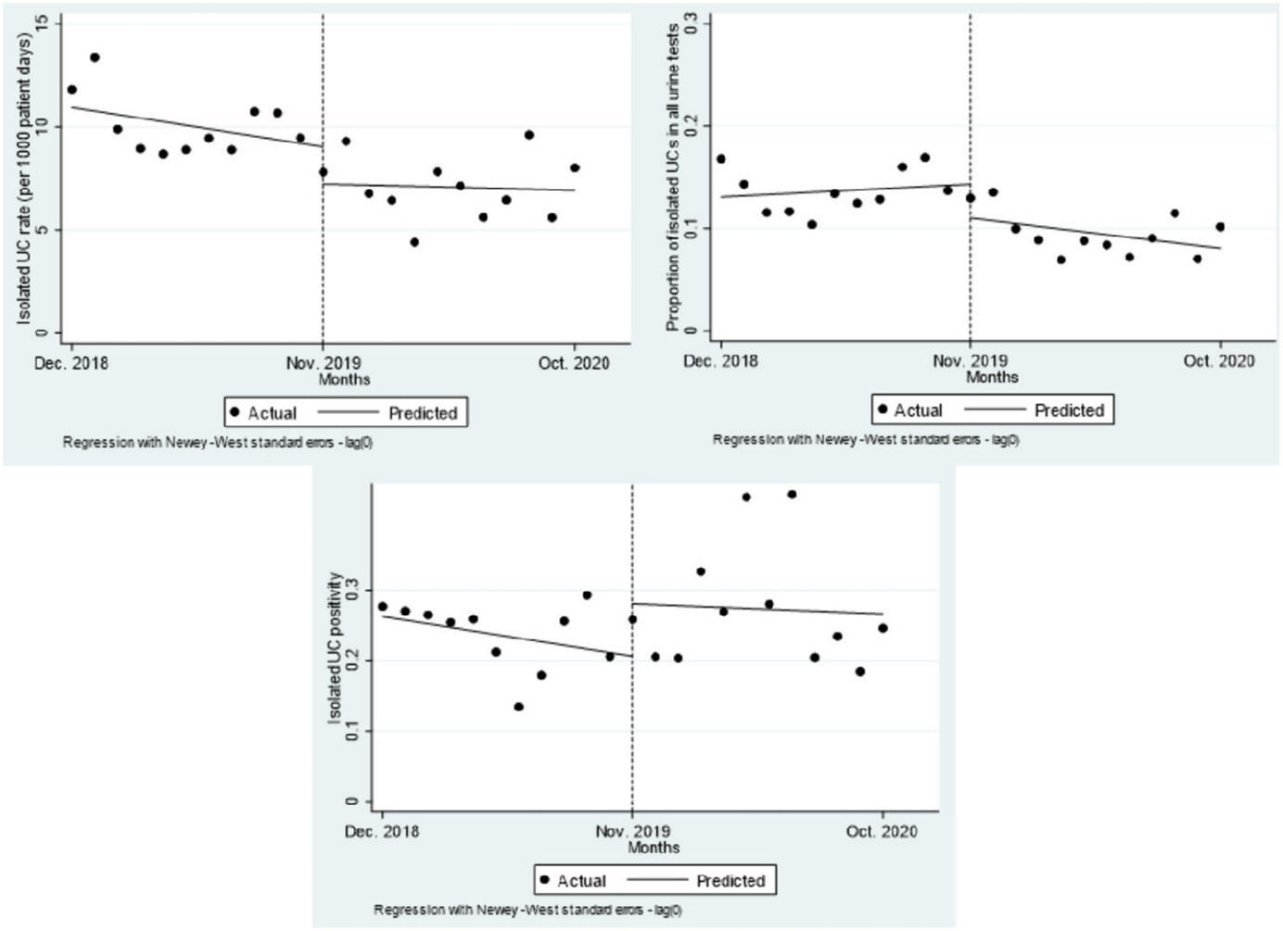
Interrupted time series analysis of isolated urine culture outcomes. Top left: Isolated urine culture rate per 1,000 patient days. Top right: Proportion of isolated of urine cultures by all urine testing ordered. Bottom left: Culture positivity. Dotted line represents time of intervention with study start date of December 1, 2018, and end date of October 31, 2020.

The interrupted time series analysis, represented graphically in Figure 1 (complete interrupted time series analysis results are reported in Supplementary Table 1), revealed a nonsignificant decrease of 30.7% in isolated urine-culture rates after the intervention compared to the preintervention period: 11.2 cultures per 1,000 patient days before the intervention versus 7.8 cultures per 1,000 patient days after the intervention (*P* = .17). There was an immediate nonsignificant increase in the proportion of testing sent as an isolated urine culture among all urine tests performed (13.1% preintervention vs 15.5% postintervention, *P* = .59), followed by a nonsignificant postintervention decrease of 2.7% per month observed (*P* = .07). No change in isolated urine culture positivity occurred: 26.9% preintervention versus 26.8% postintervention (*P* = .99).

### Indication review

A review of selected indications demonstrated that most orders did not have an indication for isolated culture testing selected, with 71.9% of the indications left blank by providers (Table 3). The next most commonly selected indications were “other” (16%), neutropenia (4.4%), and pregnancy (5.7%). A subset of the 661 tests performed in the postintervention period was randomly selected for chart review to evaluate for the appropriateness of isolated urine-culture testing and agreement between selected indication and clinical condition. Of the 100 charts reviewed, 10% had documented symptoms consistent with a urinary tract infection, and 15% received antibiotics for a presumed urinary tract infection with only 6% of those having symptoms consistent with a urinary tract infection. In the review, 67% did not have documentation of a condition for which isolated urine culture would be appropriate. Of those with selected indications, there was agreement between selected indication and presence of appropriate clinical condition in only 8% of the reviewed charts.

**Table 3.** Indications for Isolated Urine-Culture Testing

Indication, No. (%)	Total (N=661), No. (%)
Neutropenia	29 (4.4)
Pregnancy	38 (5.7)
Pre-urologic surgery	4 (0.6)
Sample volume inadequate to perform UA in preterm neonates only	1 (0.2)
Nephrologist performed UA	2 (0.3)
None of the above (Select UARC)	6 (0.9)
Other	106 (16.0)
Blank	475 (71.9)

Note. UA, urinalysis; UARC, urinalysis with reflex to culture.

### Sensitivity analysis

Several months after the intervention was implemented, the COVID-19 pandemic affected the study center. To evaluate the potential impact on the study results, a sensitivity analysis was performed by removing data from March 1, 2020, to May 31, 2020. Overall, results were similar to those of the primary analysis (Supplementary Table 2). There was no noticeable impact on the primary outcome with this period excluded. However, in the additional outcomes assessed, the postintervention trend in proportion of testing sent as an isolated urine culture became nearly statistically significant (decrease of 3% per month, *P* = .05).

## Discussion

In this study, the implementation of a provider-selected indication requirement in the laboratory order interface for isolated urine-culture testing did not significantly decrease culture rates. Although we detected significant changes seen between intervention periods when comparing median culture rates, there was no significant difference in culture rates when analyzed using an interrupted time series analysis, which considers pre- and postintervention trends.^18^ This finding supports the strengths of utilizing an analysis method that incorporates those trends when determining the potential impact of an intervention, compared to a before-and-after analysis which does not. The proportion of all urine test orders represented by isolated urine-culture orders did not differ significantly between study periods, and neither did the culture positivity. These findings reflecting the lack of change in ordering practices after the intervention.

Previous studies on the impact of indication specification requirements for isolated urine culture testing are limited. One prior multicenter study^19^ evaluated the effect of a new laboratory order interface that included a similar indication requirement in the implemented order set. This study reported a 40% reduction in culture rates after the intervention, although only 45% of orders placed after the intervention were appropriate based on chart review.^19^ Indication selection for testing remains underutilized in testing algorithms; a survey of acute-care hospitals in the Society for Healthcare Epidemiology Network (SRN) revealed that only 17% require specification of an order indication.^20^ Although this study did not demonstrate successful reduction in testing as a result of this intervention, with specific improvements, namely removing the option to skip the selection of an indication, and possibly deleting the “other” category, this intervention has the potential to prompt appropriate testing.

Measuring appropriate use of urine testing is vital because it is one of the most frequently performed diagnostic tests in a hospital setting. One large national retrospective cohort study estimated that urine testing is performed in up to 47% of hospital admissions.^21^ Prior studies have estimated that 58%–68% of urine cultures sent in hospital settings are not for guideline-based indications,^22,23^ suggesting that this testing is frequently misused. Smaller reviews have also found similar trends, with nearly 50% of testing ordered without a specified clinical indication and almost 30% for a nonspecific indication.^24^ Our study had similar findings, with 67% of reviewed charts lacking a specific indication for isolated urine testing. Many of our study patients were tested for nonspecific findings, including broad evaluation for fever or increasing leukocytosis, and only 10% of patients had documented symptoms consistent with a urinary tract infection.

Overtreatment of asymptomatic bacteriuria represents a threat contributing to growing antimicrobial resistance. This is a frequent issue; retrospective studies having found that 32.8%–41% of patients with asymptomatic bacteriuria receive antibiotic therapy for a suspected UTI.^25,26^ Although we did find lower rates of antimicrobial prescription in asymptomatic patients than these prior studies, the ultimate goal is to eliminate treatment of asymptomatic bacteriuria altogether unless the patient meets one of the rare criteria to do so (eg, pregnant patients or those who are about to undergo invasive urologic surgery). Thus, much work remains to be done.

In our study, many providers did not select an indication for isolated urine-culture testing, despite being prompted by the laboratory order interface. The indication was left blank for 71.9% of the tests after the intervention began. Previous studies have found similar difficulties with indication selection,^19^ and the most commonly selected indication was the first indication on the list, even if not most appropriate, potentially reflecting a desire to expedite the ordering process rather than reading a longer list of indications. Although significant provider education was provided before, during, and after the intervention in our study by infectious disease physicians and infection prevention specialists, the nonsignificant results seen may reflect a need for a more strategic educational approach toward ordering providers.

Utilizing the results of the study, several next steps should be considered. First, an initial and simple intervention would be implementing a stricter algorithm that does not allow a lack of indication for ordering and eliminates the “other” indication category. Another intervention that may be considered given these results is further restriction of isolated urine testing by requiring approval by antimicrobial stewardship teams for order placement, which also allows continued provider education. A more drastic intervention that could be considered is the suppression of the reporting of urine cultures when ordered for noncatheterized patients with the option for ordering providers to request cultures be reported, which has been shown to have a strong impact on clinician behavior with a decrease in treatment of asymptomatic bacteriuria.^27^

This study had several limitations. It had a single-center design, and we did not have data on method of testing collection, such as whether the specimen was collected from a catheterized patient. Isolated culture testing is typically more common among patients with urinary catheters and patients in the intensive care units,^17^ so these data would likely provide greater understanding of provider ordering practices and potential interventions. Another potential limitation is that the study period included the onset of the COVID-19 pandemic, which may have affected patient testing, potentially by decreasing testing because hospital capacity was limited and elective procedures were suspended. The sensitivity analysis performed did not show changes in the study results when the initial pandemic period was excluded; thus, the pandemic likely did not affect the intervention. We used >100,000 CFU as a criteria for positive culture. Although standard in the literature,^28^ this parameter may have underestimated the number of true infections. Strengths of our study include that our dataset consisted of a large number of tests performed at an academic medical center with an accessible electronic ordering system, providing complete data. An additional strength was the utilization of the interrupted time series design; this design provided valuable insight into pre- and postintervention trends.

In conclusion, the addition of an indication specification requirement for isolated urine culture testing did not significantly decrease the isolated urine-culture rates at our institution. A review of the specified indications revealed that this testing was often inappropriately selected for patients. These findings highlight the need for more sophisticated diagnostic algorithms to guide providers to the most appropriate testing for their patients, with the goal of reducing treatment of asymptomatic bacteriuria. Although this study did not include a more in-depth evaluation into the driving factors causing overutilization of urine testing, the reasons are likely multifactorial including inappropriate beliefs about patient risks, practices of peers and mentors, and lack of knowledge. Gaining a better understanding of these implementation barriers is paramount because we can then design interventions to target these barriers, enabling the system to impart meaningful change.

## Supplementary material

For supplementary material accompanying this paper visit https://doi.org/10.1017/ash.2022.243. click here to view supplementary material
