# Supplementary material for: Impact of provider-selected indication requirement on urine test utilization and positivity
Source: Antimicrob Steward Healthc Epidemiol. 2022 Jun 23;2(1):e103. doi: 10.1017/ash.2022.243 (PMC9726588; doi:10.1017/ash.2022.243)
Supplement: Supplementary file 1 [file S2732494X22002431sup001.docx]

# **Impact of Provider Selected Indication Requirement on Urine Test Utilization and Positivity**

Jessica A. Penney MD^1^, Angie Mae Rodday PhD^2^, Paola Sebastiani PhD^2^, David R. Snydman MD^1^, Shira Doron MD^1^

^1^ Division of Geographic Medicine and Infectious Disease, Tufts Medical Center, Boston, Massachusetts, United States

^2^ Institute for Clinical Research and Health Policy Studies, Tufts Medical Center, Boston, Massachusetts, United States

**Supplemental Material**

Urine tests

N = 17,220

3,707 tests excluded (repeat testing during admission)

Urine tests, first test from admission

N = 13,513

697 tests excluded for patients <18 years

Adult urine tests

N = 12,816

11,322 tests excluded for tests ordered as urinalysis with reflex culture

Adult urine isolated culture tests

N = 1494

**Figure 1 Data Selection for Isolated Urine Culture Study Inclusion**: Figure demonstrates the selection process to determine testing eligible for study inclusion and analysis.

**Table 1 Interrupted Time Series (ITS) Analysis Pre- and Post-Intervention Results with Difference between Periods**

|  | Pre-Intervention | | | | | | | Post-Intervention | | | | | | | | | Difference^1^ | |
| --- | --- | --- | --- | --- | --- | --- | --- | --- | --- | --- | --- | --- | --- | --- | --- | --- | --- | --- |
|  | Beta | Exp(beta) | | | | | p | Beta | Exp(beta) | | | | | | | p | Beta | p |
| Isolated UC Rate (per 1000 patient days) | | | | | | | | | | | | | | | | | | |
| *Intercept^2^* | 2.41 | 11.2 | | | <0.001 | | | 2.045 | 7.75 | | | <0.001 | | | | | -0.37 | 0.17 |
| *Slope^3^* | -0.017 | | | 0.98 | | 0.23 | | 0.029 | 1.03 | | | | | 0.73 | | | 0.012 | 0.54 |
| Proportion of all Urine Testing (%)^4^ | | | | | | | | | | | | | | | | | | |
| *Intercept* | -2.04 | 0.13 | | | <0.001 | | | -1.88 | 0.15 | | | | <0.001 | | | | 0.15 | 0.59 |
| *Slope* | 0.01 | | 1.01 | | | 0.63 | | -0.027 | | 0.97 | | | 0.075 | | | | -0.034 | 0.11 |
| CuLture Positivity (%)^5^ | | | | | | | | | | | | | | | | | | |
| *Intercept* | -1.31 | 0.27 | | | <0.001 | | | -1.32 | 0.27 | | | | <0.001 | | | | -0.0069 | 0.99 |
| *Slope* | -0.02 | 0.98 | | | | 0.36 | | -0.0003 | | | 0.99 | | | | 0.99 | | 0.019 | 0.52 |
| ^1^ Difference calculated as post-intervention minus pre-intervention beta  ^2^ Intercept represents rate at start of pre- and post-intervention period, respectively  ^3^ Slope represents pre-and post-intervention trend, respectively  ^4^ Proportion of all urine testing measured as isolated urine culture/all urine testing (isolated urine culture + UARC)  ^5^ Culture positivity measured as culture positive for organisms/isolated culture performed | | | | | | | | | | | | | | | | | | |

**Table 2 Sensitivity Interrupted Time Series (ITS) Analysis Removing COVID-19 Impacted Months**

|  | Pre-Intervention | | | | | Post-Intervention | | | | | Difference^1^ | |
| --- | --- | --- | --- | --- | --- | --- | --- | --- | --- | --- | --- | --- |
|  | Beta | Exp(beta) | | | p | Beta | Exp(beta) | | | p | Beta | p |
| Isolated UC Rate (per 1000 patient days) | | | | | | | | | | | | |
| *IntercepT^2^* | 2.41 | 11.2 | | <0.001 | | 2.13 | 8.41 | | <0.001 | | -0.28 | 0.26 |
| *Slope^3^* | -0.017 | | 0.98 | 0.23 | | 0.008 | 0.99 | | 0.55 | | 0.025 | 0.63 |
| Proportion of all Urine Testing (%)^4^ | | | | | | | | | | | | |
| *Intercept* | -2.04 | 0.13 | | <0.001 | | -1.79 | 0.17 | | <0.001 | | 0.25 | 0.38 |
| *Slope* | 0.01 | 1.01 | | 0.62 | | -0.029 | 0.97 | | 0.05 | | -0.019 | 0.08 |
| CuLture Positivity (%)^5^ | | | | | | | | | | | | |
| *Intercept* | -1.31 | 0.27 | | <0.001 | | -1.4 | 0.24 | | <0.001 | | -0.09 | 0.83 |
| *Slope* | -0.02 | 0.98 | | 0.36 | | -0.0008 | | 1.00 | 0.97 | | 0.019 | 0.51 |
| Sensitivity analysis performed by removing March-May 2020  ^1^ Difference calculated as post-intervention minus pre-intervention beta  ^2^ Intercept represents rate at start of pre- and post-intervention period, respectively  ^3^ Slope represents pre-and post-intervention trend, respectively  ^4^ Proportion of all urine testing measured as isolated urine culture/all urine testing (isolated urine culture + UARC)  ^5^ Culture positivity measured as culture positive for organisms/isolated culture performed | | | | | | | | | | | | |
